# Form, Function and Feeding: Changes in Tooth Size and Shape Associated With Ontogenetic Changes in Prey Consumption by Australian White Sharks (*Carcharodon carcharias*)

**DOI:** 10.1002/ece3.72795

**Published:** 2026-01-26

**Authors:** Emily Hunt, Yuri Niella, Amy F. Smoothey, Ezequiel M. Marzinelli, David Raubenheimer, Russell Bradford, David J. Booth, Victor M. Peddemors

**Affiliations:** ^1^ School of Life and Environmental Sciences & Charles Perkins Centre The University of Sydney Sydney New South Wales Australia; ^2^ IMOS Animal Tracking Facility Sydney Institute of Marine Science Mosman New South Wales Australia; ^3^ New South Wales Department of Primary Industries and Regional Development Sydney Institute of Marine Science Mosman New South Wales Australia; ^4^ School of Life and Environmental Sciences The University of Sydney Sydney New South Wales Australia; ^5^ CSIRO Environment Hobart Tasmania Australia; ^6^ School of the Life Sciences University of Technology Sydney Ultimo New South Wales Australia

**Keywords:** Elliptical Fourier Analysis, feeding ecology, functional morphology, ontogenetic niche shift, shark dentition, tooth morphology

## Abstract

White sharks undergo pronounced ecological and dietary shifts across ontogeny, and their teeth play a central role in mediating these changes. Understanding the complexity within shark tooth and jaw mechanics, plus the fine‐scale tooth morphology, can provide insights into how feeding strategies and, hence, dietary niches and ecological function evolve with age and size. These morphological changes underpin ontogenetic niche shifts, revealing how functional adaptations in dentition enable white sharks to exploit different prey resources throughout development. This study provides novel insights into the ontogenetic and positional variation in *C. carcharias* dentition, integrating both Elliptic Fourier Analysis (EFA) and traditional morphometric approaches. We reveal significant patterns of tooth morphology that vary with jaw position and ontogenetic stage, reflecting functional adaptations to changing dietary and biomechanical demands. A key ontogenetic shift was identified as teeth transitioned from narrow, cuspidate forms with accessory cusplets in juveniles to broader, serrated teeth in larger individuals. We found no significant differences in tooth morphology between sexes, aligning with known similarities in diet and body shape in the eastern Australian white shark population. Significant anterior‐to‐posterior variation in tooth form was observed within the jaw, with lateral teeth becoming more compressed and recurved, suggesting functional transitions in prey handling throughout the jaw. Additionally, we documented structural changes in jaw morphology at approximately 210 cm PCL, corresponding to broader teeth and increased bite capacity. These shifts likely reflect developmental milestones in feeding capability, supporting the transition from a solely piscivorous diet to the inclusion of marine mammal prey.

## Graphical abstract

White sharks exhibit distinct ontogenetic shifts in tooth morphology that underpin changes in diet and ecological function. Using Elliptic Fourier Analysis and traditional morphometrics, we reveal significant variation in dentition across jaw position and developmental stage, with a major transition occurring at 210 cm PCL and at the sixth tooth within the jaw. These findings highlight functional adaptations in feeding strategies that support shifts from piscivory to marine mammal predation.

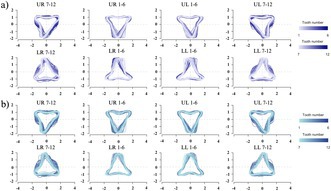

## 1 Introduction

Many organisms undergo distinct functional transitions that shape their dietary specialisation and other ecological interactions (Turner Tomaszewicz et al. 2017; Werner and Gilliam 1984; Zelditch et al. 2004), known as ontogenetic niche shifts (Werner and Gilliam 1984). Ontogenetic niche shifts are adaptively important, often representing changes in resource use with an increasing body size (Wilson 1975) or across life‐history stages, for example, from prioritising growth and survival to reproduction (Bolnick et al. 2003; Tinker et al. 2007). Niche shifts also have significant implications for community ecology, which traditionally has assumed that species are composed of identical individuals (Nakazawa 2015). It is thus important to understand and establish reliable markers of ontological niche shifts, especially in ecologically important keystone species that are difficult to observe directly, such as marine predators (e.g., large sharks; see Dedman et al. 2024).

Individual differences in diet and other factors related to foraging ecology, such as habitat use, are becoming increasingly evident in several species of sharks, which show notable differences in the allometric scaling of morphological features throughout development (Gayford et al. 2023; Hunt et al. 2024; Matich and Heithaus 2015; Seamone et al. 2024; Yun and Watanabe 2023). For example, in tiger sharks, *Galeocerdo cuvier* , the frequency of predation on turtles, marine mammals and crustaceans increases with body size (Aines et al. 2017; Dicken et al. 2017; Lowe et al. 1996). This ontogenetic variation in diet is also displayed in lemon sharks, *Negaprion brevirostris* (Newman et al. 2011), and the sevengill shark, *Notorynchus cepedianus* (Ebert 2002), revealing a shift from predominantly opportunistic benthic foraging to a more selective diet of fish and marine mammals as they grow. Furthermore, these dietary shifts are usually accompanied by morphological changes in the shape and size of the body and head (Fu et al. 2016), and the documentation of these changes has been used to highlight shifts in resource availability (Tyler‐Bonner and Horn 2000; Zelditch et al. 2004).

These morphological variations in body shape are often manifested through the allometric changes of the head shape and surrounding musculature linked to dietary shifts with age (Fu et al. 2016; Habegger et al. 2012; Huber et al. 2006). Additionally, ontogenetic changes contributing to dietary change have also been shown through the allometric scaling of the caudal fin (Hunt et al. 2024; Lingham‐Soliar 2005; Seamone et al. 2024). Increases in the surface area of the caudal fin in multiple shark species can lead to increased propulsion through the water, in turn allowing capture of fast‐moving, larger pelagic prey (Hunt et al. 2024; Irschick and Hammerschlag 2014; Lingham‐Soliar 2005).

As cosmopolitan apex predators and a species frequently involved in shark bite incidents, the white shark, *Carcharodon carcharias*, is a species of ecological, conservation and public safety importance (Domeier 2012). The white shark generally consumes a diet consisting of cephalopods, elasmobranchs and teleosts (Brown et al. 2010; Grainger et al. 2020; Jaime‐Rivera et al. 2014; Klimley 1994; Lipscombe et al. 2024; Tricas and McCosker 1984). However, throughout ontogeny, the inclusion of marine mammals increases as they transition from juveniles (< 3 m TL) to sub‐adults (> 3 m TL) (Bruce and Bradford 2012). This ontogenetic shift in diet is also accompanied by changes in habitat use, whereby young‐of‐the‐year (YOY) individuals are typically restricted to warmer, coastal nursery areas. As they grow, they become increasingly thermally tolerant and expand their range, utilising a broader spectrum of habitats, including both coastal and offshore environments (Lee et al. 2021; Spaet et al. 2020), ranging in temperatures between 2°C–30°C (Skomal et al. 2017).

Marine predators, including white sharks, show distinct biomechanical adaptations of the mouth and its associated features, including gape, bite force and tooth shape, for grabbing, gripping and dismembering prey (Cullen and Marshall 2019; Davit‐Béal et al. 2007; Ferrara et al. 2011; Riverón et al. 2025; Tricas and McCosker 1984). The teeth allow an individual to distribute prey into manageable portions and remove inedible segments of the body (Jones 2009; Whitenack et al. 2010). Historically, much of the functional analysis conducted on the feeding apparatus in sharks was focused on muscle and cranial movement (Huber et al. 2006; Motta et al. 2008); however, more recent investigations have incorporated tooth form and function in better understanding foraging ecology (French et al. 2017; Goodman et al. 2022; Riverón et al. 2025; Whitenack and Motta 2010). Furthermore, tooth morphology can provide insight into both dietary preferences and biomechanical function. Broadly, species with dietary preferences of ‘softer’ prey, such as cephalopods, typically have cuspid teeth, whereas molariform teeth are usually present in species that forage on ‘harder’ prey like crustaceans (Corn et al. 2016; Powter et al. 2010). Beyond these general categories, variation in tooth shape can further reflect functional adaptations to feeding. For example, slim, smooth teeth demonstrate a better ability for piercing and puncturing through skin, whereas wider, serrated teeth are better at slicing and cutting (Frazzetta 1988; Riverón et al. 2025). However, Whitenack and Motta (2010) conclude that shark tooth morphologies were functionally similar to each other, albeit that narrow teeth performed better at puncturing prey, whilst all tooth shapes appeared to produce similar draw capabilities.

French et al. (2017) investigated tooth height in sharks to examine changes in feeding ecology. However, focusing solely on tooth height overlooks important variations in shape that are closely tied to function and prey handling. A more detailed analysis of tooth morphology can reveal shifts in biomechanical adaptation and dietary specialisation that are not captured by linear measurements alone (Whitenack and Motta 2010), as previously demonstrated in bull sharks, *Carcharhinus leucas* (Goodman et al. 2022).

Given their broad thermal tolerance, complex ontogeny and well‐documented dietary shifts, white sharks represent an ideal species to explore how tooth morphology changes with ecological and functional pressures across life stages. Not only do these shifts reflect internal development, but, as apex predators, their ontogenetic transitions also have direct and indirect effects on prey populations and, in turn, community structure (Kim et al. 2012; Nakazawa 2015; Papastamatiou et al. 2024). Understanding how tooth morphology changes across life stages can provide a mechanistic link between form and ecological function, offering insights into how morphological traits may influence trophic roles in complex ecosystems. In this study, we therefore examined the ontogenetic changes in the tooth shape of extant white sharks and the potential influence it has on their foraging ecology. By investigating how tooth shape changes across jaw positions and throughout life stages, we aimed to examine where these morphological alterations occur and their relative importance in feeding ecology. On the basis of previous studies outlining the biomechanics of teeth (Abler 1992; Frazzetta 1988; Whitenack and Motta 2010), we predicted that the teeth will transition from a more cuspidate shape with cusplets, suitable for a diet predominantly composed of fish, to wider and thicker‐shaped teeth with large serrations for hunting mammals.

## 2 Materials and Methods

### 2.1 Sample Collection

We analysed the jaws of 97 deceased white sharks encompassing the various life stages outlined by Bruce and Bradford (2012). These stages include young‐of‐the‐year (YOY, *n =* 11), juveniles (JWS, *n =* 62), subadult (SUB, *n =* 6) and adults (MAT, *n =* 10); however, some jaws were from sharks of unknown body length (*n =* 6). The majority (76%) of samples were from white sharks caught between 1999 and 2024 in the New South Wales Shark Meshing Program (SMP) (Reid et al. 2011). The remaining samples were collected by other means, including commercial fishing operations, law enforcement confiscations and museum specimens (Table 1).

**TABLE 1:** Source and Acquisition of *Carcharodon carcharias* jaws included in this study.

Organisation	Number of jaws	Method
NSW DPIRD Fisheries	74	SMP
CSIRO	10	Commercial fishing operations/Confiscated
WA DPI Fisheries	6	Strandings/Confiscated
WA Museum	4	Donated
Rodney Fox Museum	2	Fisherman caught pre‐protection
Flinders University	1	Confiscated

### 2.2 Morphometric Measurements

**FIGURE 1:**
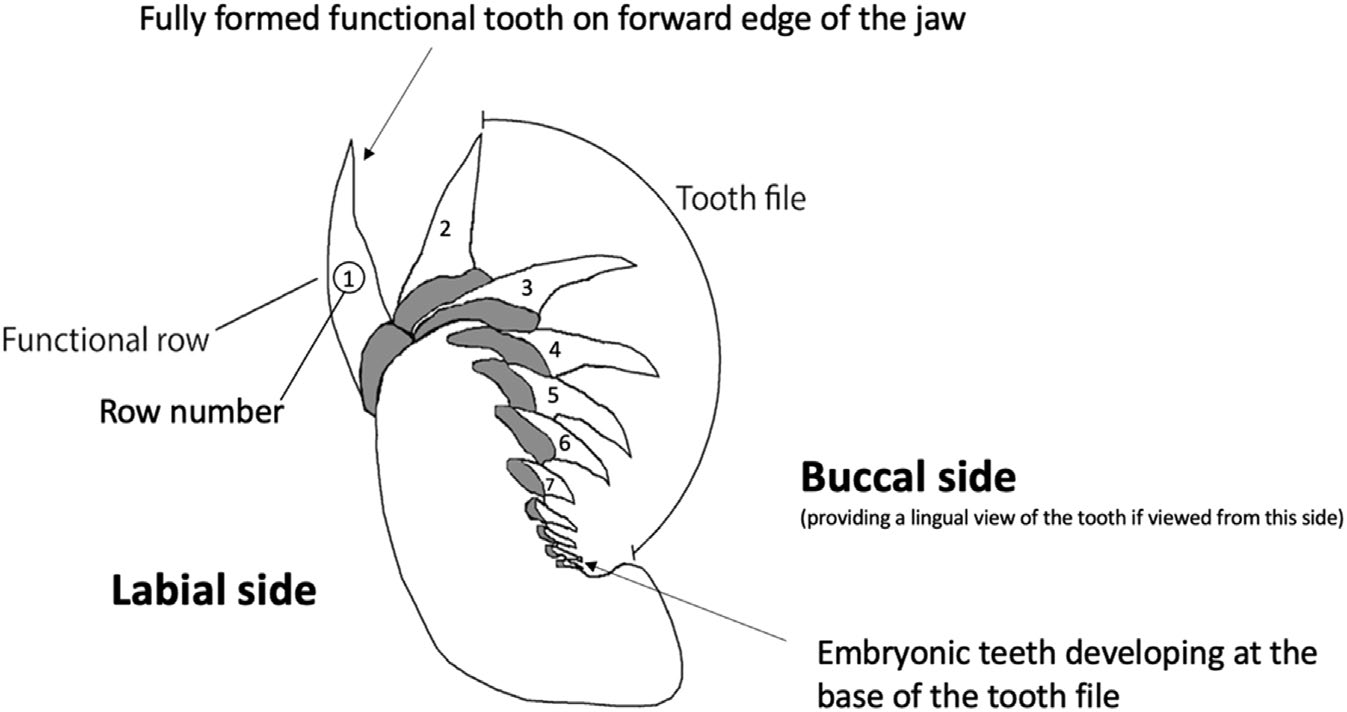
Cross‐section of the lower jaw showing the functional row of teeth and subsequent replacement teeth within the tooth file. This diagram has been adapted from Becker et al. (2000).

**FIGURE 2:**
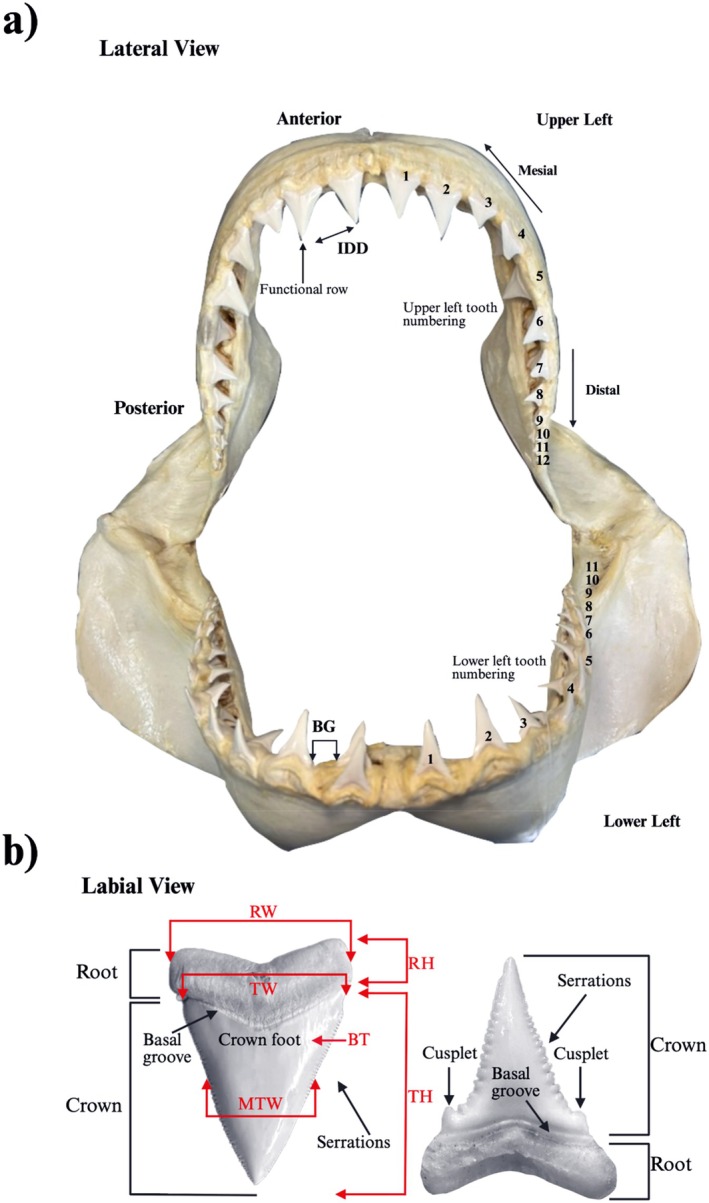
*C. carcharias* (a) jaw structure, showing the functional row of teeth measured for analysis and tooth numbering and (b) tooth structure adapted from Ebert and Stehmann (2013).

Morphometric data on teeth were collected from the jaws, using standard protocols established by NSW DPIRD. The functional row (first row) of teeth was measured for the upper and lower jaw, using the secondary tooth within the same tooth file if a functional tooth was absent or damaged (Figure 1). The first two teeth within a tooth file were assumed to be equivalent, ascribing to the fast tooth turnover rate outlined by Grainger et al. (2020). For the following measurements, jaws were divided into the upper and lower hemispheres and left and right sides (Figure 2a). The individual teeth were numbered following Moyer et al. (2015), starting from the centre of the jaw and ascending to the outer edge. The morphometric measurements collected included (a) tooth (crown) height (TH), (b) tooth width (TW), (c) the thickness at the enameloid base of the first 4 teeth on each side of the jaw (BT), i.e., the crown foot, (d) the interdental distance between the tips of the teeth (IDD), (e) the basal gap between the base of the teeth (BG), (f) and the width at the midpoint of the tooth (MTW) (Figure 2b, see Table S1 for all abbreviations). The root height (RH) and root width (RW) were also collected for the first two teeth on the right side of the mouth for the upper and lower jaw (Figure 2b). Additional measurements were also taken for the jaw using inelastic twine from the jaw joints and adapted from Lowry et al. (2009), including (a) the upper jaw circumference (UC), (b) the lower jaw circumference (LC), (c) and the jaw width (JW). To examine if there is a distinct size when the juvenile sharks discontinue developing tooth cusplets, their presence or absence was also recorded. Cusplet details were not recorded during early access to many jaws, resulting in 37 jaws from New South Wales being available for analysis for this feature. Necropsies of 91 individuals allowed for body length measurements to be made available for each animal to the nearest millimetre. Because of the increased margin of error with total length measurements of white sharks (Hunt et al. 2024), the more accurate precaudal length (PCL) was used as the ‘standard’ metric of length for the subsequent analysis.

### 2.3 Elliptical Fourier Analysis (EFA)

Photographs of all teeth in the functional rows of both upper and lower jaws were taken in situ from the labial side of the 97 white shark jaws at our disposal using an Apple iPhone 14 (Version iOS 16.3.1). To minimise parallax error, the phone was mounted in a rig positioned approximately 7 cm away from each tooth (Figure S1). Images were then edited using the Adobe Photoshop 2024 software (version 25.12.3) (Adobe 2024) to generate silhouettes of the individual teeth. The silhouettes were standardised to a 3024 × 3024 px format at 300 dpi resolution before being used for EFA. EFA was used to examine the change in tooth shape across jaw positions and throughout ontogeny. EFA describes the shape of an object by fitting a function consisting of a series of harmonics (trigonometric curves) to a set of four coordinates on the Cartesian plane (two *x* and *y* components, respectively) (Cullen and Marshall 2019). This nearest pixel approach minimises intra‐observer bias by converting the tooth outlines into a series of complex Fourier descriptors, which enables the quantification of morphological variations (Goodman et al. 2022). A greater proportion of an object's shape is described with increasing harmonics. Only seven harmonics are required to account for 99.9% of the variation in tooth shape, as these coefficients characterise the size, shape, and orientation of each harmonic ellipse (Cullen and Marshall 2019). The EFA was performed following the methods outlined by Goodman et al. (2022), using the Momocs package (Bonhomme et al. 2014). The root structure was not examined in this analysis as it was obstructed by the gums.

### 2.4 Data Analysis

To investigate variation in white shark tooth morphology with ontogeny and tooth position in the jaw, two comparative methods were used: one derived from outline‐based morphometrics using Elliptic Fourier Analysis (EFA), and the other from classic morphometric measurements. All data analysis was conducted in the R Statistical Environment (RStudio Team 2021). A paired t‐test was used to examine the bilateral symmetry of the upper and lower jaw by comparing left and right tooth counts. A two‐factor analysis of variance (ANOVA) was then applied to examine the effect of life stage and sex on tooth counts.

Each dataset was subjected to principal component analysis (PCA) to reduce dimensionality and extract the primary axes (coefficients) of variation in tooth shape (Figure S2), including all corresponding morphometric data (i.e., all measurements for traditional morphometrics and all harmonic coefficients for the EFA). The EFA coefficients were processed using PCA, and the first two principal components were extracted to capture the majority of morphological variance. This was subsequently repeated for the classic morphometrics, using five measurements per tooth to quantify the shape (excluding BT, as it was only taken for the first four teeth). PCA was performed using a correlation matrix to minimise the influence of differences in measurement variance, ensuring that no single traditional morphometric variable or EFA harmonic coefficient disproportionately affected the analysis. A scree plot was then used to confirm that the first two axes accounted for most of the variance in the data.

To ensure the variables were normally distributed, histograms were used on the extracted PCA scores to visually assess their distribution and check for any deviation (Figure S3). To evaluate the best model structure, both linear (GLMM) and non‐linear (GAMM) models were compared using the extracted PCA scores as the response variable. The Akaike information criterion (AIC) values confirmed that the Generalised Linear Mixed Model (GLMM) was more appropriate for the data. Final models using the mgcv package (Wood 2017) incorporated tooth position (UR, UL, LR, and LL), tooth number and precaudal length (PCL) as fixed effects, with individual shark ID included as a random effect (model selection Tables S2 and S3). The standardised partial residuals of the PCA coefficients were plotted against tooth number and PCL to examine points where statistically significant changes occur in the tooth shape for both the classic morphometric and EFA datasets. After identifying points of statistically significant change for both the tooth number and PCL, we used coefficient (coo) plots to visualise the shape variation using reconstructed shapes from the individual EFA coefficients, dividing the plots into the four jaw positions for each identified group.

Standardised partial residuals were also used to examine additional classic morphometric variables that were not included in the multivariate analysis. These included the base thickness, measured for the first four teeth on each side of the jaw, as these are assumed to play a key role in the initial prey capture. The root surface area (CH * CW) of tooth UR1 was calculated to evaluate structural stability, and the jaw circumference and width were measured to explore broader patterns in jaw morphology. Additionally, the TH was also examined individually using a generalised additive mixed model (GAMM) with a k value of 10 to highlight variation in the upper jaw that may be overlooked in the multivariate analysis. All models were initially run separately by sex and showed consistent trends in tooth shape changes in response to PCL and tooth number. As no difference was found between males and females in either the PCA or EFA dataset, they were therefore combined in the following analyses.

## 3 Results

### 3.1 Bilateral Symmetry

The 97 animals analysed (43 females, 38 males and 16 sex was undetermined) ranged in size from 90.85 cm PCL to 491.3 cm PCL. No significant difference was found in bilateral symmetry between the upper (mean difference = 0.11 ± 0.71 SD) and lower (mean difference = −0.14 ± 0.67 SD) left and right sides in terms of tooth counts (t(86) = 1.52, *p >* 0.05 and t(85) = −1.93, *p >* 0.05, respectively), with both hemispheres differing by a maximum of 2 teeth. Neither sex nor body length was significantly associated with tooth counts (ANOVA *F*_2,75_ = 1.56, *p >* 0.05 and *F*_3,75_ = 0.66, *p >* 0.05, respectively). Cusplets were observed in individuals up to 235 cm PCL; however, the size at which they discontinue developing varied widely, with the smallest individual lacking cusplets measuring 180 cm PCL (mean PCL = 162.1 ± 27.0 SD).

### 3.2 Classic Morphometric vs. EFA Method Comparison

The PCAs were conducted separately for the EFA coefficients and the classic morphometric variables displayed consistent patterns of shape variation, with clear separation of teeth on the basis of jaw position and a strong primary axis of morphological differentiation (Figure S2). For the EFA dataset, 55.62% of the total variation was explained by the first two principal component scores, with PC1 explaining 36.38% of the variance and PC2 explaining 19.24% (Figure S2a). The differences in shape between the upper and lower jaw hemispheres occurred primarily on the vertical plane through the *ao* and *co* coefficients, with the remaining coefficients predominantly driving the horizontal variation in the PCA scores. Conversely, 96.60% of the total shape variation was explained by the first two principal component scores in the classic morphometric dataset (Figure S2b). Within this, PC1 alone explained 83.15% of the variance, and PC2 accounted for an additional 13.45%. Similar to the EFA model, the differences in shape between the upper and lower hemispheres also occurred along the vertical axis, primarily driven by the MTW and BG. Both models showed substantial similarity between the left and right sides.

The GLMMs used to assess the influence of jaw position, tooth number and PCL on tooth shape differed between the two datasets (Tables 2 and 3). For both models, tooth number and PCL were significant predictors of variation in tooth shape (Table 2). The random effect for shark ID was also highly significant (Table 2), indicating considerable individual‐level variation. Despite these significant effects, the EFA model only explained 4.3% of the deviance, suggesting limited overall explanatory power. In contrast, the classic morphometric model showed a much stronger fit, explaining 19.8% of the deviance. Overall, the classic morphometrics captured a larger proportion of the shape variation in relation to biological and positional factors compared to the EFA coefficients.

**TABLE 2:** GLMM of *C. carcharias* tooth shape for the EFA analysis, including the effects of PCL and interactions between jaw position and tooth number.

Variable	Estimate	SE	*t*‐value	*p*
Intercept	−1.84e‐01	1.60e‐02	−11.48	< 0.001
PCL	3.50e‐04	5.50e‐05	6.36	< 0.001
LL: Tooth number	5.44e‐02	9.97e04	54.52	< 0.001
LR: Tooth number	5.16e‐02	9.81e‐04	52.60	< 0.001
UL: Tooth number	−1.34e‐02	8.73e‐04	−15.34	< 0.001
UR: Tooth number	−1.34e‐02	8.84e‐04	−15.81	< 0.001

**TABLE 3:** GLMM of *C. carcharias* tooth shape for the classic morphometric analysis, including the effects of PCL and interactions between jaw position and tooth number.

Variable	Estimate	SE	*t*‐value	*p*
Intercept	−9.64e‐02	2.81e‐02	−3.43	< 0.001
PCL	−3.16e‐03	1.19e‐04	−26.59	< 0.001
LL: Tooth number	1.32e‐01	1.68e‐03	79.19	< 0.001
LR: Tooth number	1.31e‐01	1.60e‐03	81.74	< 0.001
UL: Tooth number	1.23e‐01	1.40e‐03	87.79	< 0.001
UR: Tooth number	1.23e‐01	1.41e‐03	86.76	< 0.001

### 3.3 Tooth Shape Throughout the Jaw

**FIGURE 3:**
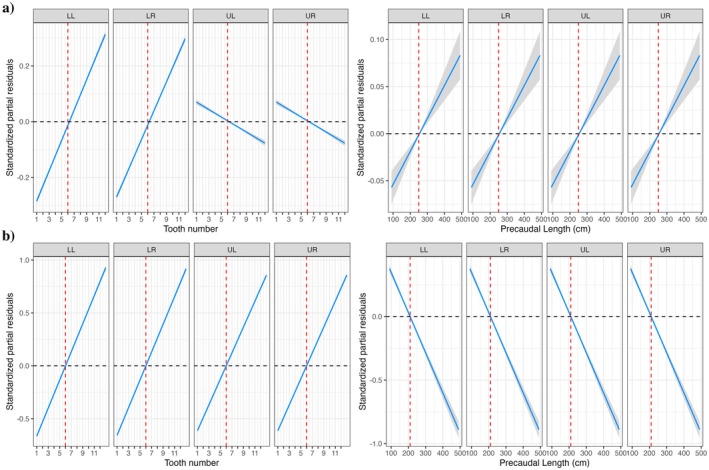
GLMM of the standardised partial residuals for (a) EFA coefficients describing tooth shape across tooth number and body length (PCL), and (b) classic morphometric coefficients describing tooth shape across tooth number and body length (PCL). Shaded bands and dashed lines indicate the 95% confidence intervals and null effects, respectively. The red dashed lines indicate the inflexion points where there is a significant shift in tooth shape.

**FIGURE 4:**
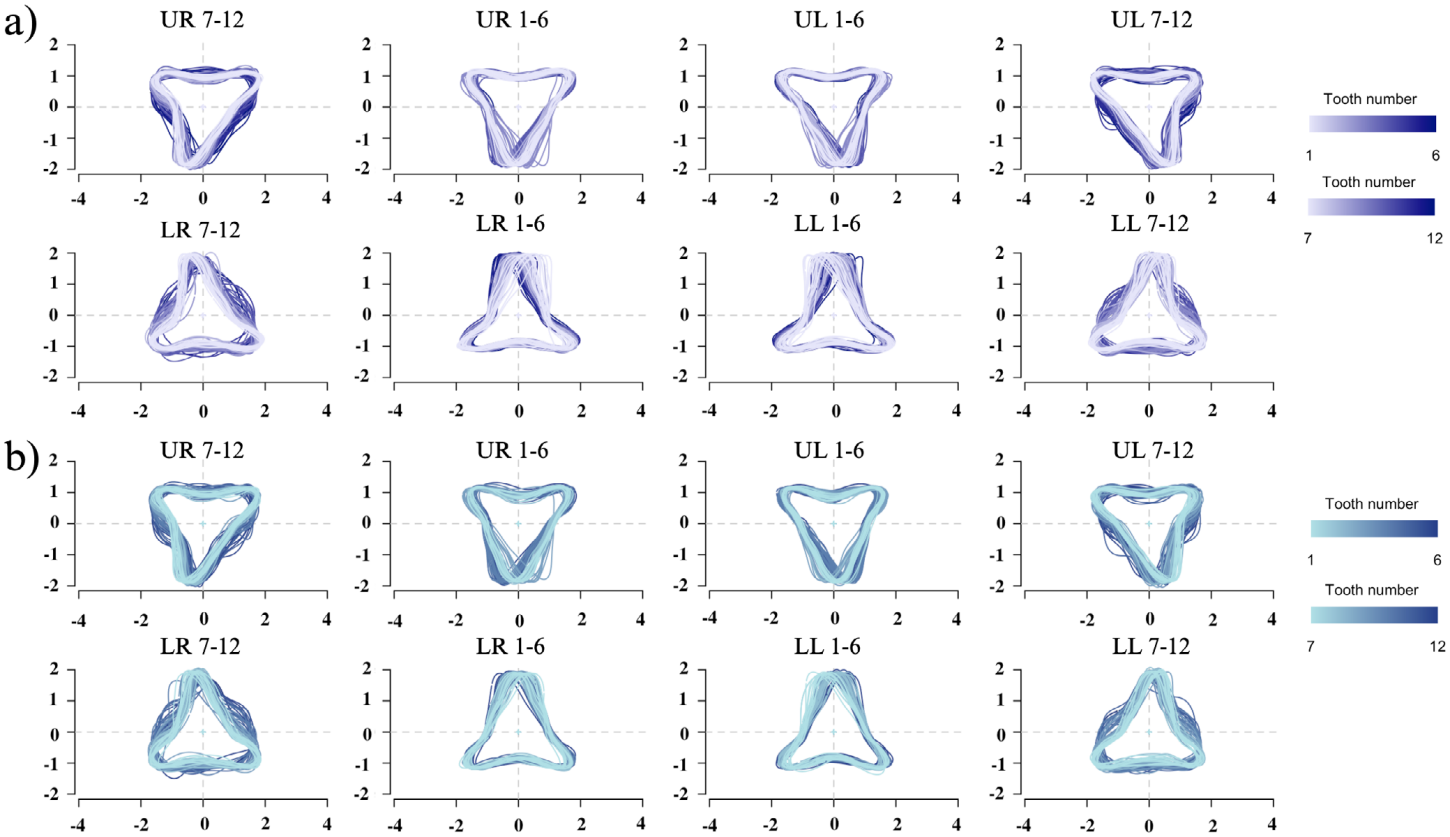
Overlaid Elliptic Fourier tooth outline reconstructions for *C. carcharias* with (a) PCL < 250 cm and (b) PCL > 250 cm. Each panel includes a sequence of six adjacent teeth per jaw quadrant. Colour‐coded overlays represent tooth position within the sequence, with lighter hues corresponding to the most mesial (central) tooth (tooth 1 or 7) and progressively darker hues indicating more distal (lateral) teeth (tooth 6 or 12).

The GLMM models for both the EFA and classic morphometric datasets both exhibited a consistent inflexion point at the 6th tooth across all jaw positions, indicating a distinct morphological transition in tooth shape around this location (Figure 3). Further investigation using overlaid elliptic Fourier tooth outline reconstructions highlights the gradual transition in tooth morphology across the jaw arcade, with teeth 1–6 presenting a more symmetrical shape and teeth 7–12 exhibiting a distally curved shape (Figure 4).

### 3.4 Tooth Shape Throughout Ontogeny

Ontogenetic changes in tooth shape were suggested by the GLMMs, with divergence occurring at different PCL depending on the morphometric method. In the classic morphometric model, residuals indicated a significant shift in shape beginning at approximately 210 cm PCL, whereas the EFA model identified this transition at a larger body size, around 250 cm PCL (Figure 3). When grouped by body size, EFA outlines from smaller sharks (PCL ≤ 250 cm) tended to show more slender and curved forms, particularly in anterior teeth. In contrast, teeth from larger sharks (PCL > 250 cm) appeared broader and more triangular (Figure 4). This trend was most pronounced in the lower jaw, with the first 6 teeth exhibiting a broader shape at PCL > 250.

### 3.5 Additional Measures

**FIGURE 5:**
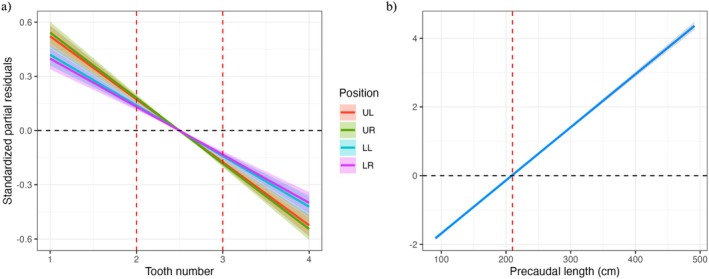
The standardised partial residuals for the BT in (a) tooth number and (b) PCL. Shaded bands and dashed lines indicate the 95% confidence intervals and null effects, respectively. The red dashed lines indicate the inflexion points where there is a significant shift in tooth shape.

**FIGURE 6:**
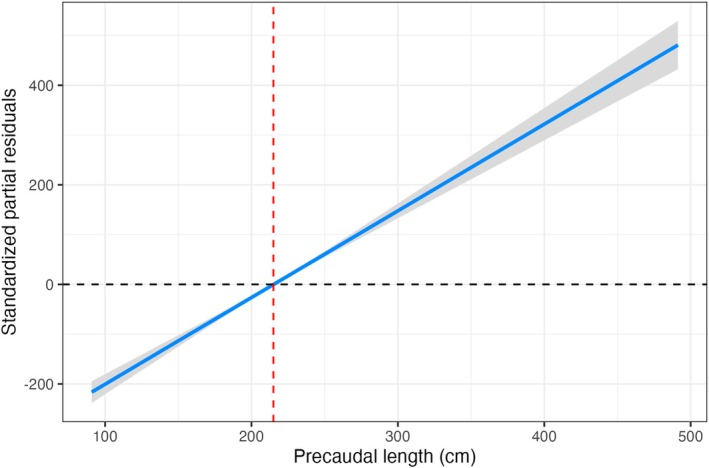
The standardized partial residuals for the root surface area using tooth UR1. Shaded bands indicate the 95% confidence intervals, respectively. The red dashed line indicates the inflection points where there is a significant shift in tooth shape.

**FIGURE 7:**
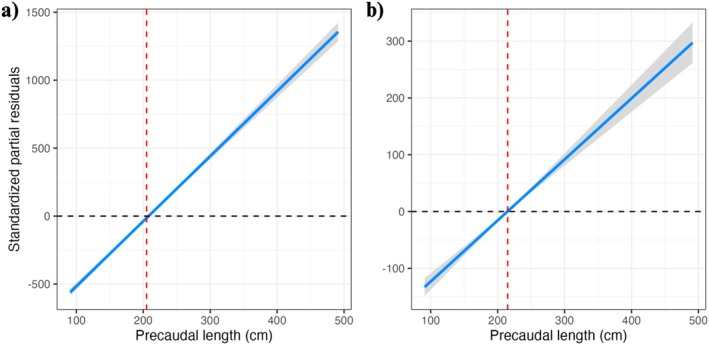
The standardized partial residuals for (a) jaw circumference and (b) jaw width with PCL for *C. carcharias* jaws. Shaded bands indicate the 95% confidence intervals, respectively. The red dashed lines indicate the inflection points where there is a significant shift in tooth shape.

**FIGURE 8:**
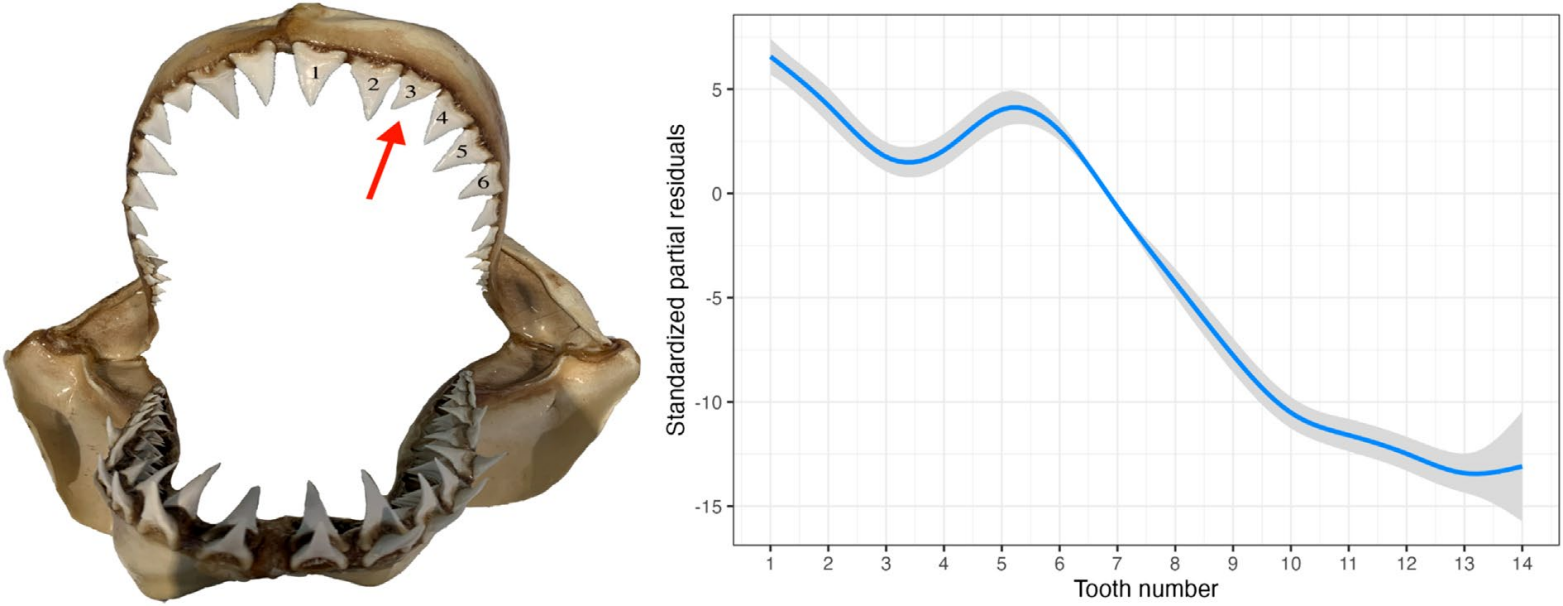
The standardized partial residuals for the TH across tooth number for the upper jaw hemisphere to highlight the relative difference in tooth 3 and 4. Shaded bands indicate the 95% confidence intervals, respectively.

The standardised partial residuals from the additional linear models examined similarly revealed clear ontogenetic shifts in the classic morphometric variables not included in the primary multivariate PCA. The residuals for the BT displayed a significant shift at approximately 210 cm PCL (Figure 5b). However, the distinct change in BT occurred after the second tooth, suggesting structural reinforcement in anterior teeth, possibly during the transition to larger prey (Figure 5a). Similarly, the root surface area of tooth UR1 also exhibited a distinct increase at 210 cm PCL (Figure 6), likely reflecting the need for enhanced tooth stability in larger individuals. The broader jaw metrics followed comparable patterns to the changes in tooth shape. Jaw circumference showed a significant increase relative to body length at approximately 205 cm PCL (Figure 7a), whereas jaw width increased more gradually, with a similar inflexion point at around 210 cm PCL (Figure 7b). However, there was greater variability observed in the jaw width at larger PCL, as shown by the broader confidence intervals in the residuals. Finally, an analysis of the TH alone revealed that upper hemisphere teeth three and four are relatively shorter than their neighbouring teeth (Figure 8a).

## 4 Discussion

This study provides the most comprehensive morphometric analysis to date of tooth shape variation in white sharks, revealing significant patterns associated with tooth position within the jaw and throughout ontogeny. By using both EFA and traditional morphometrics, our results highlight the complex interplay of biological, developmental, and ecological factors that shape tooth morphology in this apex predator, offering insights into the functional implications of these variations (Wainwright et al. 2004; Wainwright 2007). We observed a strong bilateral symmetry in tooth counts and tooth shape between the left and right sides of the jaw. This symmetrical structure likely promotes an even distribution of mechanical forces during feeding, reducing stress on the jaw and improving prey handling efficiency (Dean et al. 2005; Ferrara et al. 2011; Gomes et al. 2011; Wroe et al. 2008).

The discontinued development of cusplets in animals > 235 cm PCL highlights the functional role cusplets play in juveniles to enhance grasping ability and prey retention when feeding on smaller, mucilaginous prey and provide an adaptive advantage during early life stages (Bemis et al. 2015; Herman et al. 1991; Perez et al. 2018) when the Australasian white shark diet predominantly comprises smaller teleost prey (Grainger et al. 2020). However, there is considerable inter‐individual variability, as the smallest individual lacking cusplets was only 180 cm PCL. This size corresponds with the findings of Hussey, Christiansen, et al. (2012); Hussey, MacNeil, et al. (2012), who observed the initial appearance of marine mammals in the diet of South African white sharks at 194 cm PCL, potentially suggesting that early reductions in cusplet expression may coincide with the onset of a dietary transition toward including larger, more robust prey. Previous research has documented sexual dimorphism in white shark tooth shape, with males exhibiting broader upper first teeth and greater distal inclination in the upper third teeth during growth (French et al. 2017). In contrast, our data showed no significant differences in tooth shape between sexes. This aligns with the findings of Grainger et al. (2020), who found no dietary differences between female and male eastern‐Australian white sharks and suggests that the sex of an individual is unlikely to influence tooth morphology in this species. Similarly, Hunt et al. (2024) found no significant sexual dimorphism in the body shape of Australasian white sharks, further supporting our lack of sexual differences in this study of white shark teeth.

Interestingly, our findings differed from those of French et al. (2017), who relied on two‐dimensional photographs, whereas our study used outline‐based morphometrics and multiple classic morphometric measurements of actual teeth throughout the entire jaws of white sharks to quantify the shape. Our findings confirm that they are variable across the jaw. The tooth shape varied significantly by tooth number, with a clear anterior‐to‐posterior gradient in both the upper and lower jaw. Upper teeth exhibited broader, more triangular crowns with greater curvature compared to the slender, piercing‐shaped teeth observed in the lower jaw, especially in juvenile sharks. The consistent differences observed between the upper and lower jaw hemispheres align with the known feeding behaviour of white sharks, whereby lower teeth are primarily used for prey capture and retention and upper teeth for sawing and dismemberment (Compagno 2001; Frazzetta 1988; Purdy 1996; Whitenack et al. 2010).

The overlaid elliptic Fourier tooth outline reconstructions displayed a clear pattern of mediolateral heterodonty evident across the functional tooth row. This was further examined through the standardised partial residuals in the GLMM, highlighting a pronounced shift in morphology at the sixth tooth. This represents a major inflexion point along the jaw, beyond which teeth become considerably more laterally compressed and recurved. This gradation may be indicative of a transition from an impaling or cutting function in anterior teeth to a shape better suited for shearing or tearing in more lateral teeth (Herman et al. 1991; Shimada 2002). These patterns reflect a high degree of within‐row (sequential monognathic) heterodonty, a condition where adjacent teeth differ significantly in form, as described by Zimm et al. (2025). Although some shark species display considerable variation between upper and lower jaws without pronounced differences between adjacent teeth, white sharks exhibit both (Zimm et al. 2025), supporting the notion that heterodonty may be functionally adaptive.

The individual analysis of the tooth height in the upper jaw revealed the distinctive morphology of the third and fourth teeth, in particular their smaller size and slight angling toward the second tooth. Although these subtle features were not fully captured in the PCA, they warrant further investigation as they may offer greater insight into functional adaptations within the jaw. For example, the slight angling of the teeth may indicate a specific role in prey retention, potentially used as a specialised tool in hunting pinnipeds (French et al. 2017; Martin et al. 2005). The relative size of the third and fourth tooth, on the other hand, is likely influenced by the underlying cranial morphology, as these teeth align with a region of the upper jaw where key tendons associated with the olfactory system are anchored (Denton et al. 2018; Staggl et al. 2022; Yopak et al. 2015), potentially constraining tooth development in this area. Additionally, the individual analysis of the base thickness in the first four teeth revealed comparable results for body length, where a distinct change in thickness occurred at 210 cm PCL. Conversely, the major inflexion point was observed after the second tooth, implying that this increased tooth thickness in the front two teeth on each side of the jaw may indicate they are the primary teeth involved in the initial bite, as they represent teeth with enhanced strength (Lucifora 2001; Siversson 1992).

One key ontogenetic trend observed in this study is the overall transition from a more cuspidate shape tooth with accessory cusplets in smaller individuals to broader, more triangular teeth with serrations in larger individuals. This trend is particularly evident in the lower jaw, whereby the shape is considerably wider at the mid‐point of the tooth in individuals > 250 cm PCL. The standardised partial residuals in the GLMM further revealed a distinct shift in tooth shape at approximately 210 cm PCL in the classic morphometry method and 250 cm PCL in the EFA method. Our results further corroborate the findings of Tricas and McCosker (1984), who found a change in tooth shape in white sharks at approximately 300 cm TL (~240 cm PCL, Hunt et al. 2024). These morphological changes demonstrate clear ontogenetic shifts in tooth shape that coincide with known changes in white shark diet and foraging behaviour. Previous studies have shown that juveniles rely primarily on a piscivorous diet (Grainger et al. 2020; Kerr et al. 2006), requiring teeth optimised for grasping and holding, whereas mature animals (> 300 cm TL) increasingly consume marine mammals, requiring broader, serrated teeth capable of slicing through dense flesh and bone (Estrada et al. 2006; Tricas and McCosker 1984). As larger prey pose a greater risk of injury during predation, successful attacks require the immobilisation of prey within 1 min (Martin et al. 2005). As such, this shift in tooth shape to broader, serrated teeth in larger individuals may enhance the initial bite to incapacitate the prey quickly, reducing both the overall handling time and the risk of injury. The increasing complexity and surface area of the teeth in larger individuals likely enhance the efficiency of processing large‐bodied, high‐calorie prey such as marine mammals (Riverón et al. 2025). These ontogenetic changes in tooth morphology therefore appear to reflect adaptive responses to mechanical and dietary demands at different life stages (Grainger et al. 2020; Hussey, Christiansen, et al. 2012; Hussey, MacNeil, et al. 2012).

Moreover, our findings also highlight substantial ontogenetic changes in jaw structure that are closely linked to functional shifts in feeding capacity and biomechanics. Notably, the standardised partial residuals indicated a distinct change in the jaw circumference at 205 cm PCL, a size supported by a jaw width increase at 210 cm PCL. This suggests a developmental shift in jaw structure, enabling support for larger, broader teeth and facilitating the transition to larger, more robust prey. Jaw width, in particular, exhibited greater variation in individuals larger than 300 cm PCL, though some of this variation may be influenced by the way specimens have been preserved. These morphological changes are accompanied by a significant increase in the surface area of the tooth root at 210 cm PCL, possibly reflecting the early stages of jaw mineralisation and structural reinforcement (Ferrara et al. 2011). White sharks are unique among elasmobranchs in possessing up to five extra layers of mineralised cartilage in the jaw, a trait acquired as they mature (Dingerkus et al. 1991). This added mineralisation reduces stress in the load‐bearing outer layers of the jaw, thereby improving its mechanical resistance to bite forces (Ferrara et al. 2011; Wroe et al. 2008). Ferrara et al. (2011) observed this mineralisation of the jaw in the transitional stages in ontogeny (~150–300 cm TL). However, the full mineralisation did not occur until sharks exceeded 300 cm TL, complementing our findings of tooth reinforcement in the surface area of the root at 210 cm PCL (~260 cm TL, Hunt et al. 2024).

Biomechanical studies show that serrated teeth in larger individuals improve efficiency by reducing shear and tear stresses when feeding (Frazzetta 1988; Riverón et al. 2025; Whitenack and Motta 2010; Whitenack et al. 2010; Wroe et al. 2008). However, this adaptation comes with trade‐offs as serrations impede puncturing, limiting effectiveness on small or hard‐bodied prey. One limitation in our dataset is that some functional teeth, particularly from large specimens, may show worn serrations because of feeding history, making serration morphology a less reliable metric for ontogenetic comparison. Although we did not quantify the serration morphology in this study, we recommend that it be included in future research to better understand its role in feeding biomechanics and ontogeny. More broadly, shark feeding systems are highly integrated, and recent syntheses highlight how tooth form, jaw kinematics, cranial musculature and cartilage mineralisation interact to shape feeding performance across ontogeny and species (Huber et al. 2019). Considering this wider functional framework, the coordination of jaw expansion, tooth reinforcement and mineralisation of jaw cartilage collectively enhances bite performance and dietary breadth as sharks grow, supporting their shift from piscivory to macropredation.

Our study provides strong evidence that white shark tooth shape is associated with both positional and ontogenetic factors, reflecting adaptations to changing dietary needs and jaw mechanics. Specifically, the slender, narrow teeth observed in juveniles likely facilitate grasping and holding of slippery teleost prey, whereas the broader, serrated teeth in adults are better suited for cutting and processing marine mammal tissue. The use of both elliptic Fourier descriptors and traditional linear measures provided complementary insights. Although linear morphometrics effectively captured gross size and proportional changes, Fourier analysis revealed subtler, holistic shape differences that may be overlooked in traditional methods. The consistency in patterns across both datasets supports the robustness of our findings and highlights the utility of combining shape quantification techniques in studies of dental functional morphology. However, the classic morphometrics captured a larger proportion of the shape variation in relation to biological and positional factors compared to the EFA coefficients and presented a greater overall explanatory power. By linking form to function across multiple scales, this work contributes to a more nuanced understanding of the ecological and evolutionary forces shaping apex predator feeding morphology.

## Author Contributions

**Emily Hunt:** conceptualization (equal), data curation (lead), formal analysis (lead), investigation (equal), methodology (equal), project administration (lead), visualization (lead), writing – original draft (lead), writing – review and editing (lead). **Yuri Niella:** data curation (equal), formal analysis (equal), investigation (equal), methodology (equal), supervision (equal), writing – review and editing (equal). **Amy F. Smoothey:** conceptualization (equal), data curation (equal), methodology (equal), supervision (equal), writing – review and editing (equal). **Ezequiel M. Marzinelli:** supervision (equal), writing – review and editing (equal). **David Raubenheimer:** conceptualization (equal), resources (equal), supervision (equal), writing – review and editing (equal). **Russell Bradford:** data curation (equal), investigation (equal), resources (equal), writing – review and editing (equal). **David J. Booth:** formal analysis (supporting), writing – review and editing (equal). **Victor M. Peddemors:** conceptualization (equal), data curation (equal), investigation (equal), methodology (equal), resources (equal), supervision (lead), writing – review and editing (equal).

## Funding

The authors have nothing to report.

## Conflicts of Interest

The authors declare no conflicts of interest.

## Supporting information


**Data S1:** ece372795‐sup‐0001‐Supinfo.docx.

## Data Availability

The data supporting the findings of this study are publicly available via the Zenodo Digital Repository: https://doi.org/10.5281/zenodo.16741930. All data are released under the Creative Commons Attribution 4.0 International (CC BY 4.0) licence.
