# Supplementary material for: Form, Function and Feeding: Changes in Tooth Size and Shape Associated With Ontogenetic Changes in Prey Consumption by Australian White Sharks (Carcharodon carcharias)
Source: Ecol Evol. 2026 Jan 26;16(1):e72795. doi: 10.1002/ece3.72795 (PMC12834646; doi:10.1002/ece3.72795)
Supplement: Supplementary file 1 — Data S1: ece372795‐sup‐0001‐Supinfo.docx. [file ECE3-16-e72795-s001.docx]

**SUPPLEMENTARY DATA**

**Table S1: Abbreviations**

| **Term** | **Abbreviation** |
| --- | --- |
| Tooth height | TH |
| Tooth width | TW |
| Tooth base thickness | BT |
| Interdental distance | IDD |
| Basal gap | BG |
| Tooth Midwidth | MTW |
| Root height | CH |
| Root width | CW |
| Upper jaw circumference | UC |
| Lower Jaw circumference | LC |
| Jaw width | JW |
| Precaudal length | PCL |
| Elliptical Fourier analysis | EFA |
| Upper right | UR |
| Upper left | UL |
| Lower right | LR |
| Lower left | LL |

**Table S2:** Stepwise model selection procedure for the GLMM of biological traits influencing the classic morphometrics method of *C. carcharias* tooth morphology. Included are all candidate models, their AICc values, ΔAIC (difference from the best model), and AIC weights (wAIC). Also shown are p-values from ANOVA comparing each model to the previous one in the sequence.

| **Model Description** | **AICc** | ΔAIC | **wAIC** | **p-Value** |
| --- | --- | --- | --- | --- |
| Null | 217124.2 | 10826.97 | <0.0001 | - |
| + PCL | 217117.8 | 10820.63 | <0.0001 | <0.01 |
| + Tooth number | 206368.0 | 70.77 | <0.0001 | <0.001 |
| + Position | 216753.7 | 10456.50 | <0.0001 | - |
| + Tooth number + PCL | 206361.1 | 63.88 | <0.0001 | - |
| + Tooth number:Position | 206304.2 | 7.00 | 0.029 | <0.001 |
| + Tooth number:Position + PCL * | 206297.2 | 0.0 | 0.971 | <0.01 |

* Final Model

**Table S3:** Stepwise model selection procedure for the GLMM of biological traits influencing the EFA method of *C. carcharias* tooth morphology. Included are all candidate models, their AICc values, ΔAIC (difference from the best model), and AIC weights (wAIC). Also shown are p-values from ANOVA comparing each model to the previous one in the sequence.

| **Model Description** | **AICc** | ΔAIC | **wAIC** | **p-Value** |
| --- | --- | --- | --- | --- |
| Null | 691802.8 | 9321.6 | <0.0001 | - |
| + PCL | 691802.9 | 9321.7 | <0.0001 | <0.001 |
| + Tooth number | 691627.4 | 9146.2 | <0.0001 | <0.0001 |
| + Position | 690592.3 | 8111.1 | <0.0001 | <0.0001 |
| + Tooth number + PCL | 691627.4 | 9146.2 | <0.0001 | <0.0001 |
| + Tooth number:Position | 682481.2 | 0.0 | 0.427 | <0.0001 |
| + Tooth number:Position + PCL * | 682481.2 | 0.0 | 0.427 | - |

* Final Model


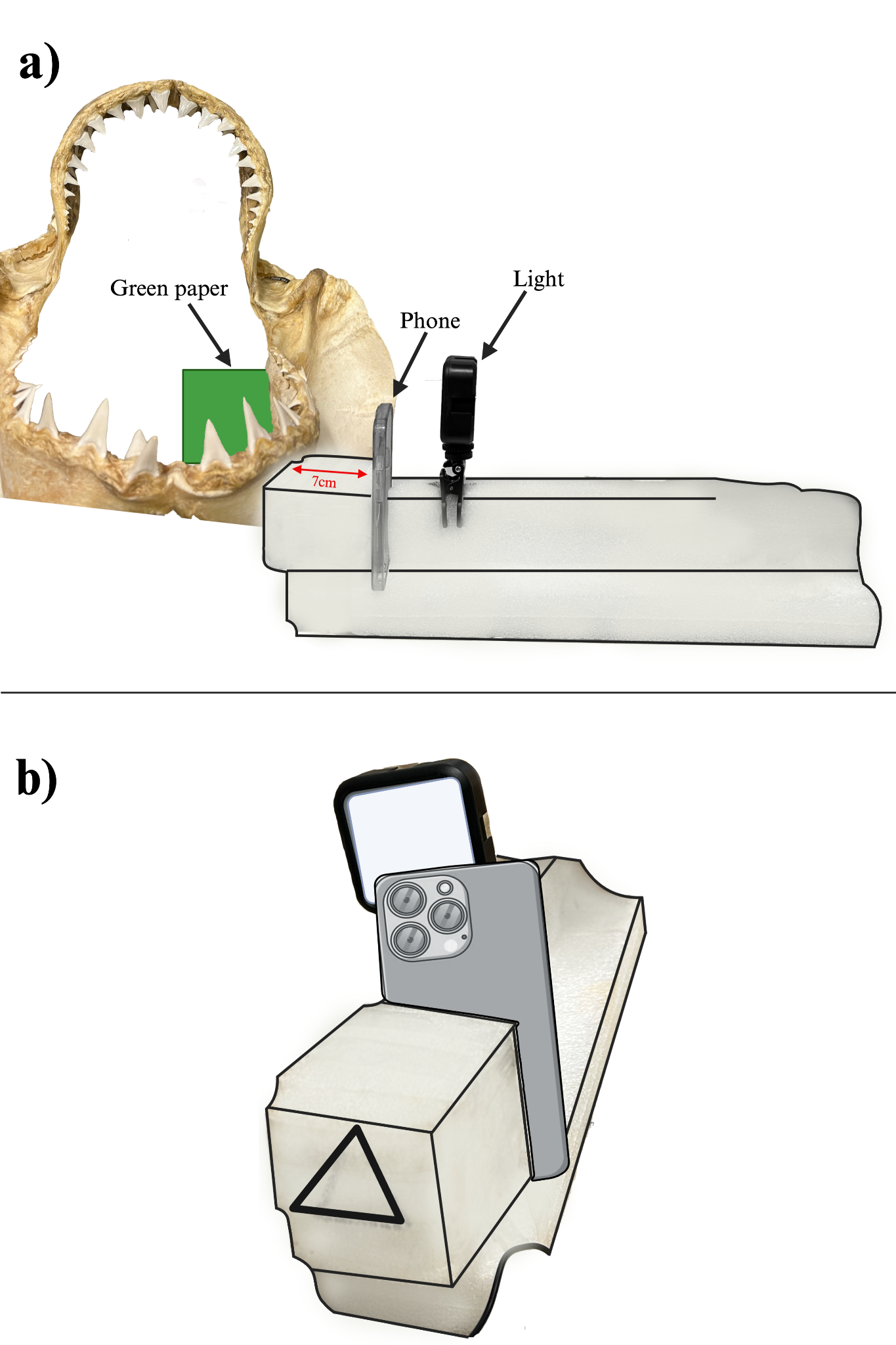


**Fig. S1** Illustrated diagram of the EFA rig setup

**
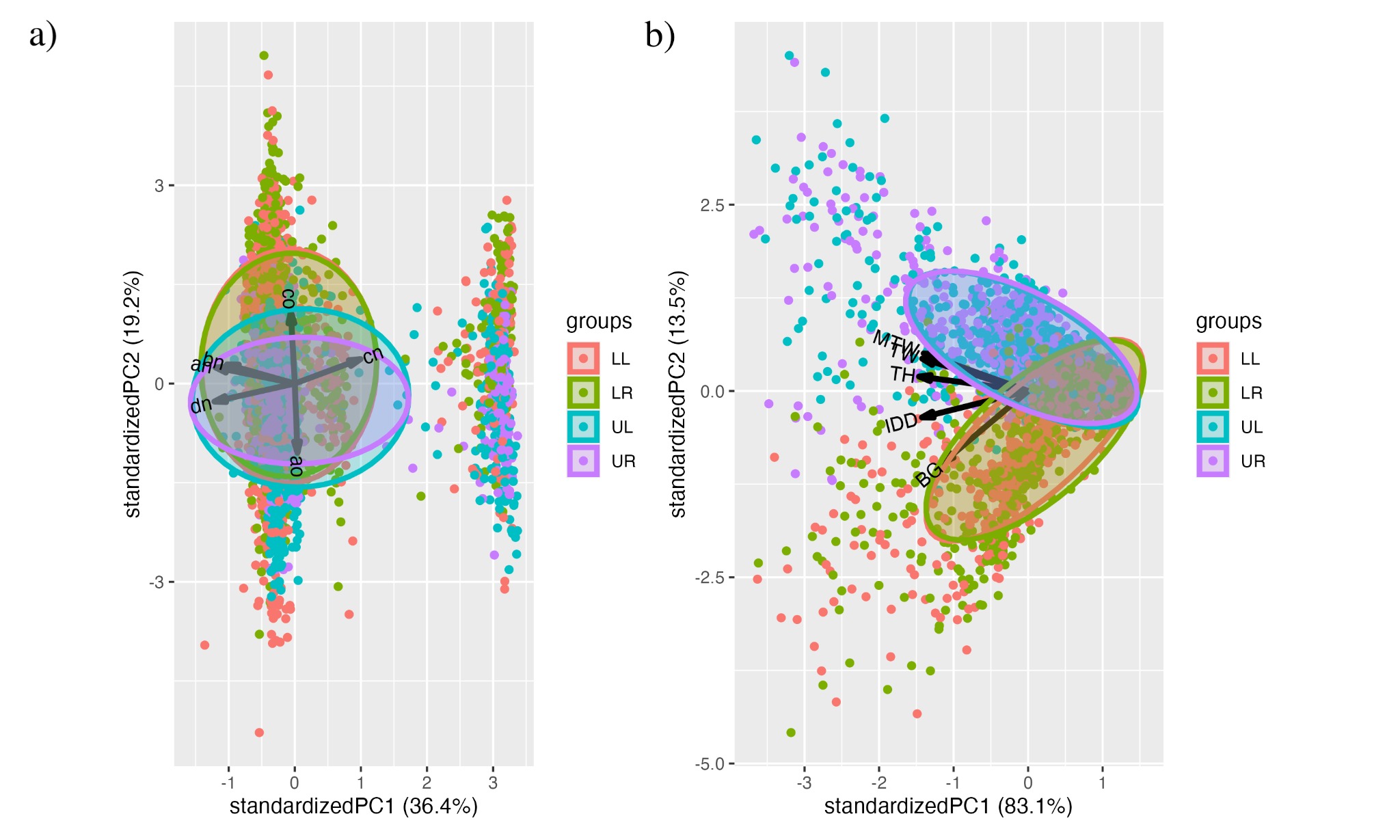
**

**Fig. S2** PCA of a) EFA coefficients and (b) classic morphometric coefficients used to observe the primary axes (coefficients) of variation in tooth shape.

**
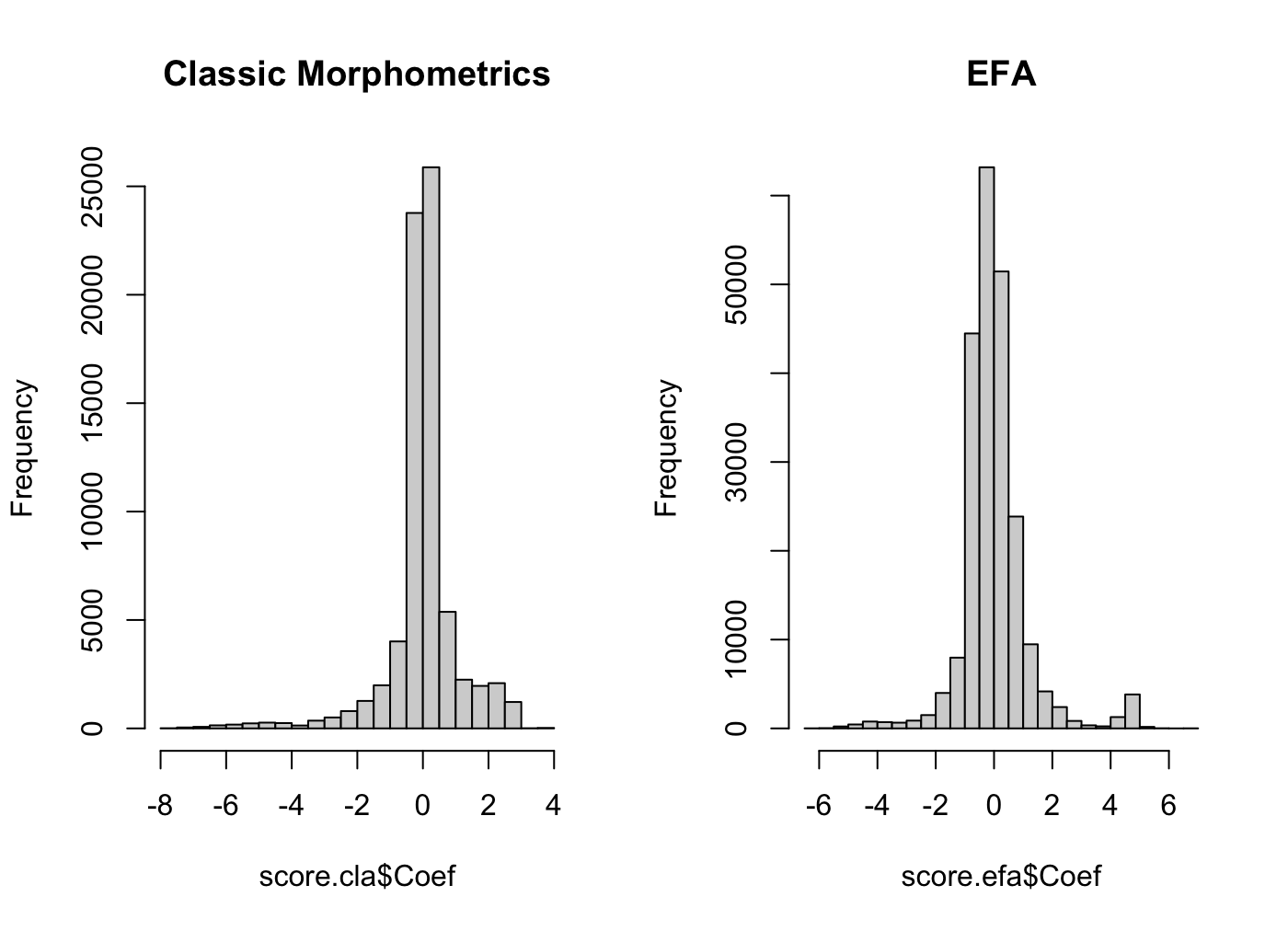
**

**Fig. S3** Histograms of the a) classic morphometric coefficients and (b) EFA coefficients returned from the respective PCA analyses.
